# Mitigation of Aflatoxin B_1_ Hepatoxicity by Dietary *Hedyotis diffusa* Is Associated with Activation of NRF2/ARE Signaling in Chicks

**DOI:** 10.3390/antiox10060878

**Published:** 2021-05-30

**Authors:** Ling Zhao, Jiang Deng, Zi-Jian Xu, Wan-Po Zhang, Mahmoud Mohamed Khalil, Niel Alexander Karrow, Lv-Hui Sun

**Affiliations:** 1 Department of Animal Nutrition and Feed Science, College of Animal Science and Technology, Huazhong Agricultural University, Wuhan 430070, China; zling@mail.hzau.edu.cn (L.Z.); JiangDeng@webmail.hzau.edu.cn (J.D.); zijianxu@webmail.hzau.edu.cn (Z.-J.X.); 2 Department of Veterinary Pathology, College of Veterinary Medicine, Huazhong Agricultural University, Wuhan 430070, China; zwp@mail.hzau.edu.cn; 3 Animal Production Department, Faculty of Agriculture, Benha University, Cairo 13736, Egypt; mahmoud.khalil@fagr.bu.edu.eg; 4 Department of Animal Biosciences, University of Guelph, Guelph, ON N1G2W1, Canada; nkarrow@uoguelph.ca

**Keywords:** *Hedyotis diffusa*, aflatoxin B_1_, hepatotoxicity, NRF2/ARE signaling, broilers

## Abstract

The objective of this study was to explore the mechanism of *Hedyotis diffusa* (HD) in mediating the detoxification of aflatoxin B_1_ (AFB_1_)-induced hepatic injury in chicks. A total of 144 one-day-old male broilers (Cobb 500) were randomly assigned to four treatment groups (*n* = 6 cages/diet, 6 chicks/cage). After three days of acclimation, the broilers were fed either a control diet (Control), Control plus 0.5 mg/kg of AFB_1_, or Control plus 0.5 mg/kg AFB_1_ with 500 or 1000 mg/kg HD for two weeks. Both serum and liver were collected at the end of the feeding trial for biochemistry, histology, and NF-E2-related nuclear factor 2 (NRF2)/antioxidant response element (ARE) signaling analysis. Compared with Control, the AFB_1_ treatment caused liver injury and decreased (*p* < 0.05) body weight gain, feed intake, feed conversion ratio, and serum albumin and total protein by 6.2–20.7%. AFB_1_ also induced swelling, necrosis, and severe vacuolar degeneration in chicks’ livers. Notably, HD supplementation at 500 and 1000 mg/kg mitigated (*p* < 0.05) the alterations induced by AFB_1_. HD supplementation alleviated (*p* < 0.05) AFB_1_-induced impairment in hepatic glutathione peroxidase activity, protein carbonyl, and exo-AFB_1_-8,9-epoxide (AFBO)–DNA concentrations by 57.7–100% and increased (*p* < 0.05) the activities of superoxide dismutase and catalase by 23.1–40.9% more than those of AFB_1_ treatment alone. Furthermore, HD supplementation at the two doses upregulated (*p* < 0.05) NRF2, NAD(P)H: quinone oxidoreductase-1, heme oxygenase-1, glutathione cysteine ligase catalytic subunit, and glutathione-S transferase A2 and A3 in livers relative to the AFB_1_ group by 0.99–3.4-fold. Overall, dietary supplementation of HD at a high dose displayed better protection effects against aflatoxicosis. In conclusion, a dietary HD supplementation at 500 and 1000 mg/kg protected broilers from AFB_1_-induced hepatotoxicity, potentially due to the activation of NRF2/ARE signaling in the chicks.

## 1. Introduction

Aflatoxins (AFs) are secondary metabolites of the fungi *Aspergillus flavus* and *Aspergillus parasiticus* that can be detected in various agricultural commodities [1,2]. Aflatoxin B_1_ (AFB_1_) is considered the most toxic variant among AFs and their metabolites. It exhibits harmful teratogenic, mutagenic, and hepatotoxic effects on both humans and livestock [3,4,5]. Moreover, AFB_1_ is also classified as a Group I carcinogen [6]. The biotransformation pathways of AFB_1_ represent the main reason for its toxicity. The bioactivation of AFB_1_ in the liver by cytochrome P450 (CYP450), a member of the phase I metabolizing enzymes, into the highly reactive exo-AFB_1_-8,9-epoxide (AFBO) [5,7] forms adducts with DNA and proteins, resulting into cytotoxicity, mutations, and DNA lesions [5,8]. On the contrary, detoxification of AFBO can occur in conjunction with glutathione (GSH), forming a non-toxic adduct that can be catalyzed by glutathione-S transferases (GST), the phase II detoxification enzymes [8]. Moreover, AFB_1_ also can induce the generation of reactive oxygen species (ROS), leading to oxidative stress and oxidation of DNA, proteins, and lipids [6,9,10]. Several strategies have been proposed to detoxify and deactivate these toxins in order to decrease their harmful effects on animals. Lately, the use of natural active biological compounds derived from plants has been attracting more attention. Some metabolites of herbs have been proven to be an effective alternative that can ameliorate the adverse effects of AFB_1_ in broiler diets [11,12,13].

*Hedyotis diffusa* (HD), a natural medicinal plant, is composed of flavonoids, iridoids, triterpenes, anthraquinones, alkaloids, lignans, coumarins, cerebrosides, and sterols [11]. These chemical compounds provide HD with multiple biological properties including antioxidant, anti-inflammatory, antitumor chemo-preventive, proapoptotic, and anti-angiogenic effects [14,15]. Since it possesses these properties, HD has been widely applied for the treatment of inflammation-related diseases, bronchitis, urethritis, and appendicitis and has been proposed as a potential therapy for liver, breast, lung, colon, and pancreatic cancers [14,15,16,17,18,19].

Interestingly, HD has shown the ability to protect against AFB_1_-induced mutation and carcinoma by inhibiting CYP450-mediated bioactivation of AFB_1_ to toxic AFBO [20,21]. Numerous natural compounds protect against toxicity of xenobiotics through regulating NF-E2-related nuclear factor 2 (NRF2)/antioxidant response element (ARE) pathway [22]. NRF2 binds to ARE, which promotes the transcriptional expression of phase II-detoxifying enzymes and antioxidant genes, such as GST, superoxide dismutase (SOD), glutathione peroxidase (GPX), catalase (CAT), glutathione cysteine ligase catalytic subunit (GCLC), heme oxygenase-1 (HO1), and NAD(P)H: quinone oxidoreductase-1 (NQO1) [23,24,25]. To our knowledge, no information is available about HD’s potential to influence NRF2/ARE signaling in the context of AFB_1_-induced hepatoxicity. Therefore, the present study was conducted to investigate whether the dietary supplementation of HD has any effect in mitigating AFB_1_-induced hepatotoxic effects through the regulation of the NRF2/ARE signaling pathway in chicks.

## 2. Materials and Methods

### 2.1. Birds, Dietary Treatments, and Sample Collections

The animal trial was conducted following the protocol approved by the Institutional Animal Care and Use Committee of Huazhong Agricultural University, China. A total of 144 one-day-old male broiler chickens (Cobb 500) were randomly assigned to 4 experimental groups, with 6 replicates of 6 chicks/cage. Chicks were fed a corn/soybean-based diet (BD) formulated to meet the nutritional requirements of broilers (Supplemental Table S1) [10] with unlimited access to water. After 3 days of adaptation, chicks in the four experimental groups were fed either the BD diet (Control), Control plus 0.5 mg AFB_1_/kg (AFB_1_), Control plus 0.5 mg AFB_1_/kg and 500 mg HD/kg (AFB_1_ + 500HD), or Control plus 0.5 mg AFB_1_/kg and 1000 mg HD/kg (AFB_1_ + 1000HD). The HD was added as the form of the extract of HD plant, which was prepared as previously described [26]. Briefly, the dried whole plant (1000 g) of HD was extracted with 70% ethanol at room temperature for 2 days and repeated three times. Then, it was vacuum dried and given 81.5 g of HD extract. The AFB_1_ was bought from Sigma Chemical Co. (St. Louis, MO, USA). The experimental duration was chosen on the basis of previous studies that reported that dietary consumption of >100 μg AFB_1_/kg ≥ 2 weeks induced hepatotoxicity in broilers [10,27]. The experiment lasted for 2 weeks, and body weights and feed intake were measured weekly. At the end of the feeding trial, 1 broiler from each replicate (thus, 6 broilers from each treatment group) were slaughtered for blood and liver sample collection for serologic and liver histologic examination, as previously described [10]. The liver samples were washed with ice-cold isotonic saline, divided into aliquots, snap-frozen in liquid nitrogen, and stored at −80 °C until use [28].

### 2.2. Serum Biochemical and Histologic Analysis

The serum activities of aspartate aminotransferase (AST) and alanine aminotransferase (ALT), and the concentrations of total bilirubin (TBIL), total protein (TP), and albumin (ALB) were measured by an automatic biochemistry analyzer (Beckman Synchron CX4 PRO). The liver tissues were examined microscopically after being fixed in 10% neutral buffered formalin, embedded in paraffin, sectioned at 5 μm, and stained with hematoxylin and eosin [29].

### 2.3. Hepatic Antioxidant Parameters and AFBO–DNA Adduct Concentrations Analysis

The activities of SOD, GPX, and CAT, along with concentrations of malondialdehyde (MDA) and protein carbonyl (PC), were measured by specific assay kits (A001, A005, A007–1, A003, and A087–1–2) purchased from the Nanjing Jiancheng Bioengineering Institute of China. Protein concentrations were measured by the bicinchoninic acid assay. The hepatic AFBO–DNA adduct concentrations were analyzed as previously described [10]. Briefly, hepatic genomic DNA was extracted by a DNA isolation kit (Tiangen Biotech Co., Ltd., Beijing, China). Then, approximately 15 μg of the genomic DNA was used to determine the AFBO–DNA adduct concentration using an AFBO–DNA adduct competitive ELISA Kit (Cell Biolabs, Inc., San Diego, CA, USA).

### 2.4. Real-Time qPCR and Western Blot Analyses

Real-time q-PCR analyses of the pertaining samples were conducted as previously described [10]. Primers (Supplemental Table S2) for the NRF2/ARE signaling-related genes and the reference gene β-actin were designed using Primer Express 3.0 (Applied Biosystems). The 2^−ddCt^ method was used for the quantification with β-actin as a reference gene, and the relative abundance was normalized to the Control group. Western blot analyses of the pertaining samples were performed as previously described [30]. The primary antibodies used for each gene product are presented in Supplementary Table S3. Protein concentrations were measured by the bicinchoninic acid assay.

### 2.5. Statistical Analysis

The data were analyzed by one-way ANOVA using SPSS statistics software (version 19, IBM) with the significance level declared at *p* < 0.05, and the Tukey–Kramer method was used for multiple mean comparisons. The data are presented as means ± standard errors (SE).

## 3. Results

### 3.1. Growth Performance, Serum Biochemistry, and Liver Histology

**Figure 1:**
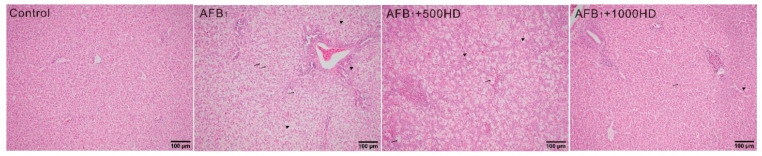
Influence of dietary AFB_1_ and HD on the histopathology of livers of chicks. The liver sections were stained with hematoxylin and eosin; Photo-micrographs are shown at 200× magnification. Here, arrows indicate swelling and necrosis; arrowheads indicate vacuolar degeneration. Control, base diet; AFB_1_, base diet supplement with 0.5 mg/kg AFB_1_; AFB_1_ + 500HD, base diet supplement with 0.5 mg/kg AFB_1_ plus 500 mg/kg HD; AFB_1_ + 1000HD, base diet supplement with 0.5 mg/kg AFB_1_ plus 1000 mg/kg HD.

No differences (*p* ≥ 0.05) were observed for the initial body weight of broilers among the four groups (Table 1). After two weeks of experimental treatments, AFB_1_ decreased (*p* < 0.05) the final body weight, body weight gain, feed intake, and gain/feed efficiency of chicks by 6.2–12.3% (Table 1). These changes induced by AFB_1_ were significantly mitigated (*p* < 0.05) by HD supplementation at 500 and 1000 mg/kg. Furthermore, although AFB_1_ did not alter (*p* *≥* 0.05) serum ALT and AST activities and TBIL concentration, it decreased (*p* *<* 0.05) ALB and TP concentrations by 16.6–20.7% relative to the Control (Table 2). Interestingly, dietary supplementation with HD at 500 and 1000 mg/kg alleviated (*p* *<* 0.05) AFB_1_-induced changes on these biochemistry variables. Moreover, the histological results indicated that AFB_1_ induced liver damage including swelling, necrosis, severe vacuolar degeneration, and bile duct hyperplasia (Figure 1). However, the hepatic injury induced by AFB_1_ was alleviated by dietary HD supplementation in a dose-dependent manner.

### 3.2. Hepatic Antioxidant Variables and AFBO–DNA Adduct Concentration

**Figure 2:**
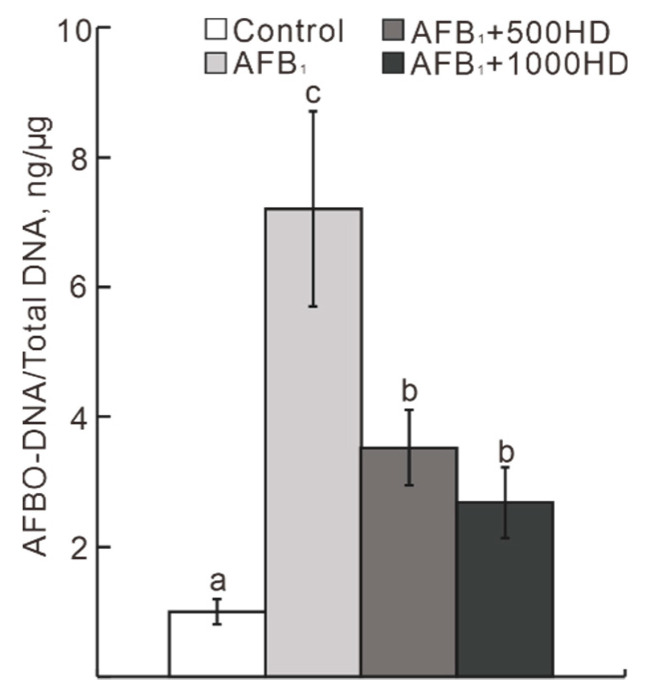
Effects of dietary AFB_1_ and HD on the concentrations of AFBO–DNA adducts in the liver of chicks. Values are means ± SEs, *n* = 6. Means for a given gene not sharing a common letter are significantly different, where *p* < 0.05. AFBO, exo-AFB1-8,9-epoxide; Control, base diet; AFB_1_, base diet supplement with 0.5 mg/kg AFB_1_; AFB_1_ + 500HD, base diet supplement with 0.5 mg/kg AFB_1_ plus 500 mg/kg HD; AFB_1_ + 1000 HD, base diet supplement with 0.5 mg/kg AFB_1_ plus 1000 mg/kg HD.

Birds exposed to AFB_1_ treatment for two weeks showed significant alterations in the hepatic antioxidant variables, as presented in Table 3. Compared to the Control, AFB_1_ decreased (*p* < 0.05) the activity of GPX (19.8%) but increased the concentration of PC (33.4%) in the liver of broilers. Notably, these changes induced by AFB_1_ were significantly mitigated (*p* < 0.05) by the dietary supplementation of HD at 500 and 1000 mg/kg. Interestingly, the activities of SOD and CAT in the liver were increased (*p* < 0.05) by 23.1–40.9% in the AFB_1_ + 1000HD group compared to the AFB_1_ group. However, the concentration of MDA in the liver was not affected (*p* *≥* 0.05) by AFB_1_ or HD. Additionally, AFB_1_ increased (*p* < 0.05) the concentration of AFBO–DNA adducts by 11-fold in the liver compared with the Control (Figure 2). Notably, dietary supplementation with HD at 500 and 1000 mg/kg reduced (*p* < 0.05) the concentration of AFBO–DNA adducts by 51.0–62.7% in comparison to the AFB_1_-supplemented group.

### 3.3. Expression of the NRF2/ARE Signaling-Related Genes

**Figure 3:**
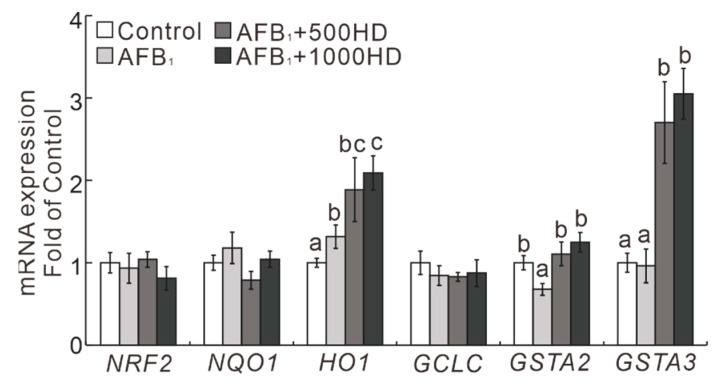
Effects of dietary AFB_1_ and HP on the relative mRNA abundances of NRF2–ARE signaling in the livers of chicks. Values are means ± SEs, *n* = 6. Means for a given gene not sharing a common letter are significantly different, where *p* < 0.05. *NRF2*, NF-E2-related nuclear factor 2; *NQO1*, NAD(P) H: quinone oxidoreductase-1; *HO1*, heme oxygenase-1; *GCLC*, glutathione cysteine ligase catalytic subunit; *GST*, glutathione-S transferase. Control, base diet; AFB_1_, base diet supplement with 0.5 mg/kg AFB_1_; AFB_1_ + 500HD, base diet supplement with 0.5 mg/kg AFB_1_ plus 500 mg/kg HD; AFB_1_ + 1000HD, base diet supplement with 0.5 mg/kg AFB_1_ plus 1000 mg/kg HD.

**Figure 4:**
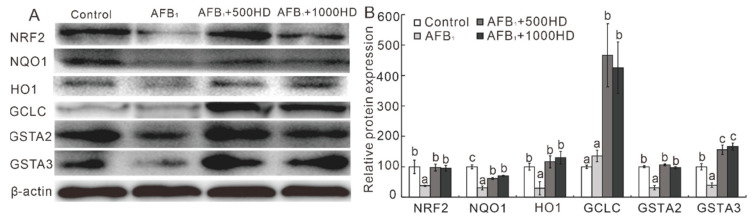
Effects of dietary AFB_1_ and HP on the relative protein abundances of NRF2–ARE signaling in the livers of chicks. For a given protein, the band was a representative image of 3–5 independent analyses (**A**), and the value shows the relative density of protein bands (mean ± SEs, *n* = 3–5; (**B**)). Means for a given protein not sharing a common letter are significantly different, where *p* < 0.05. NRF2, NF-E2-related nuclear factor 2; NQO1, NAD(P) H: quinone oxidoreductase-1; HO1, heme oxygenase-1; GCLC, glutathione cysteine ligase catalytic subunit; GST, glutathione-S transferase; Control, base diet; AFB_1_, base diet supplement with 0.5 mg/kg AFB_1_; AFB_1_ + 500HD, base diet supplement with 0.5 mg/kg AFB_1_ plus 500 mg/kg HD; AFB_1_ + 1000HD, base diet supplement with 0.5 mg/kg AFB_1_ plus 1000 mg/kg HD.

Among the six NRF2/ARE signaling-related genes examined in the liver of chicks, three genes were influenced by AFB_1_ and HD treatment (Figure 3). Specifically, AFB_1_ upregulated (*p* < 0.05) the mRNA levels of HO1 and downregulated (*p* < 0.05) the mRNA level of *GSTA2*. Interestingly, dietary supplementation of HD at 500 and 1000 mg/kg increased (*p* < 0.05) the mRNA levels of HO1, GSTA1, and GSTA3 compared to the AFB_1_ treatment group. Moreover, Western blot results showed that the hepatic protein levels of NRF2, NQO1, HO1, GCLC, GSTA2, and GSTA3 were affected by AFB_1_ and HD treatment (Figure 4). Specifically, AFB_1_ downregulated (*p* < 0.05) the protein levels of NRF2, NQO1, HO1, GSTA2, and GSTA3 in the liver of broilers. Notably, these changes induced by AFB_1_ were significantly mitigated (*p* < 0.05) by the dietary supplementation of HD at 500 and 1000 mg/kg. Strikingly, the protein levels of GSTA3 and GCLC were much higher (*p* < 0.05) in the AFB_1_ + 500HD and AFB_1_ + 1000HD groups compared to the Control and AFB_1_ groups.

## 4. Discussion

The current study showed that HD was able to mitigate hepatotoxicity induced by AFB_1_ in broilers. Birds that consumed AFB_1_ diets exhibited a reduction in final body weight, body weight gain, feed intake, and gain/feed of broilers, all of which remain in agreement with previous studies [13,31,32]. This poor growth performance could be related to anorexia and inhibition of lipogenesis and protein synthesis [32,33]. Meanwhile, AFB_1_ also induced the typical clinical and pathological signs of hepatic injury, including decreased concentrations of ALB and TP in serum as well as swelling, necrosis, severe vacuolar degeneration, and bile duct hyperplasia in the liver of broilers and Japanese quail [29,31,34]. Intriguingly, dietary supplementation of HD at 500 and 1000 mg/kg alleviated AFB_1_-induced toxic effects on performance, serum biochemistry, and hepatic histopathology. Notably, dietary supplementation with HD at the 1000 mg/kg displayed better mitigation based on these parameters than 500 mg/kg. These findings are similar to previous studies that showed that dietary supplementation of HD can mitigate AFB_1_-induced growth retardation and hepatic damage [20,21].

AFB_1_ dysregulation of the response of hepatic redox parameters (GPX and PC) indicated that the chicks exposed to AFB_1_ suffered from oxidative stress in the present study. Specifically, AFB_1_ reduced the antioxidant enzyme GPX activity and induced the protein oxidation maker PC [10,27,32]. These outcomes are similar to previous studies [10,27,32], which explained that AFB_1_ can induce oxidative stress and results in proteins, lipids and DNA oxidation, thereby causing cytotoxicity and genotoxicity [9,10,32]. Interestingly, dietary supplementation with HD at 500 mg/kg mitigated AFB_1_-induced changes in these redox parameters in the liver. Moreover, dietary supplementation of HD at 1000 mg/kg even increased the hepatic antioxidant enzymes activities of SOD, GPX, and CAT compared to levels in the AFB_1_ groups. Taken together, these outcomes indicate that HD could alleviate AFB_1_-induced oxidative stress in the liver of chicks. These findings are similar to previous studies, which have reported that HD could detoxify other xenobiotic-induced oxidative stress both in vivo and in vitro [16,35,36].

A novel finding from the current study is that the alleviation of AFB_1_-induced hepatotoxicity by HD was associated with the activation of NRF2/ARE signaling in broilers. Although the mRNA of NRF2 was not affected by AFB_1_ treatment, the NRF2 protein was upregulated by the HD treatment in the liver of broilers. As the NRF2-targeted antioxidative genes, NQO1 and HO1 played important roles in the protection against oxidative stress [25,37,38]. AFB_1_ sharply downregulated approximately 70% abundances of these two proteins, which might promote AFB_1_-induced oxidative injury in the liver of broilers. These findings are similar to previous reports [39,40]. Intriguingly, dietary supplementation of HD at 500 and 1000 mg/kg mitigated AFB_1_-induced downregulation of NQO1 and HO1 proteins, which may be attributed to reduced oxidative injury in livers induced by AFB_1_. Moreover, the phase II metabolizing enzymes GSTA2, GSTA3, and GCLC, which play roles in the detoxification of AFB_1_ by catalyzing the conjugation of toxic AFBO with GSH [8,10,27,41], can be transcriptional activated by NRF2 [23,24,25]. Similar to previous studies, AFB_1_ decreased the mRNA and protein levels of GSTA2 and GSTA3 in the liver, which may be contributed to its induced hepatic injury [8,42,43,44]. Notably, dietary supplementation with HD at 500 and 1000 mg/kg inhibited the AFB_1_-induced reduction of GSTA2 and GSTA3 proteins and increased the GCLC protein, which could help to ameliorate the AFB_1_-induced hepatotoxicity of chicks. Indeed, as the primary toxic adduct of AFBO [10,45], AFBO–DNA was sharply decreased in the liver of broilers exposed to AFB_1_ by the HD dietary supplementation. These results indicate that the protective effects of HD against AFB_1_ toxicity are potentially mediated by activating the pivotal GSTs isozymes that promote the detoxification of the highly toxic AFBO.

Paradoxically, the abundance of hepatic proteins NRF2, NQO1, HO1, and GCLC did not correlate well with their mRNA abundance. Additionally, as the target genes of NRF2, the mRNA levels of NQO1, HO1, and GCLC also did not change as well as expected. These divergences may be attributed to complex feedback or posttranscriptional mechanisms regulating the synthesis of these proteins [28,32].

## 5. Conclusions

In summary, the present study has illustrated that dietary HD supplementation could mitigate poor growth performance and hepatic injury induced by AFB_1_ in broilers, as illustrated by the amelioration of the changes in serum biochemistry and histopathologic lesions. The protective mechanism of HD against AFB_1_-induced hepatoxicity is associated with the activation of NRF2/ARE signaling in broiler chicks. Specifically, (1) HD could mitigate AFB_1_-induced oxidative stress by increasing the expression of the proteins with antioxidant capacities and (2) HD could effectively promote the expression of phase II detoxification enzymes, which play crucial roles in the detoxification of AFB_1_ by catalyzing the conjugation of highly toxic AFBO with GSH. These findings support the use of HD to prevent aflatoxicosis in chicks.

## Figures and Tables

**Table 1:** Effects of dietary aflatoxin B_1_ and HD on growth performances of broilers ^1^.

	Control	AFB_1_	AFB_1_ + 500HD	AFB_1_ + 1000HD
Initial Body Weight, g	69.4 ± 0.1	69.2 ± 0.1	69.3 ± 0.1	69.4 ± 0.2
Final Body Weight, g	647± 8.3 ^c^	575 ± 12.5 ^a^	592 ± 17.4 ^ab^	619 ± 8.8 ^b^
Body Weight Gain, g	577 ± 8.3 ^c^	506 ± 12.5 ^a^	523 ± 17.4 ^ab^	550 ± 8.8 ^b^
Feed Intake, g	676 ± 9.8 ^b^	632 ± 12.9 ^a^	636± 16.7 ^ab^	662± 8.8 ^ab^
Gain/Feed, g/kg	854 ± 9 ^b^	801 ± 24 ^a^	822 ± 17 ^ab^	831 ± 5 ^a^

^1^ Values are means ± SEs, *n* = 6. Means in a row not sharing a common letter are significantly different, where *p* < 0.05. HD, *Hedyotis diffusa*; Control, base diet; AFB_1_, base diet supplement with 0.5 mg/kg AFB_1_; AFB_1_ + 500HD, base diet supplement with 0.5 mg/kg AFB_1_ plus 500 mg/kg HD; AFB_1_ + 1000 HD, base diet supplement with 0.5 mg/kg AFB_1_ plus 1000 mg/kg HD.

**Table 2:** Effects of dietary aflatoxin B1 and HD on serum biochemistry of broilers ^1^.

	Control	AFB_1_	AFB_1_ + 500HD	AFB_1_ + 1000HD
ALT, U/L	3.40 ± 0.43	2.73 ± 0.55	3.79 ± 0.56	3.23 ± 0.72
AST, U/L	268 ± 18.7	270 ± 27.4	230 ± 5.8	243 ± 21.7
TBIL, µmol/L	3.64 ± 0.06	3.65 ±0.08	3.70 ± 0.15	3.75 ±0.2
ALB, g/L	16.3 ± 0.5 ^b^	14.1 ± 0.9 ^a^	17.6 ± 1.7 ^ab^	16.6 ± 0.5 ^b^
TP, g/L	26.1 ± 1.2 ^b^	20.7 ± 3.0 ^a^	23.8 ± 2.0 ^ab^	37.5 ± 5.0 ^c^

^1^ Values are means ± SEs, *n* = 6. Means in a row not sharing a common letter are significantly different, where *p* < 0.05. ALB, albumin; ALT, alanine aminotransferase; AST, aspartate aminotransferase; HD, *Hedyotis diffusa*; TBIL, total bilirubin; TP, total protein; Control, base diet; AFB1, base diet supplement with 0.5 mg/kg AFB1; AFB1+500HD, base diet supplement with 0.5 mg/kg AFB1 plus 500 mg/kg HD; AFB1+1000HD, base diet supplement with 0.5 mg/kg AFB1 plus 1000 mg/kg HD.

**Table 3:** Effects of dietary aflatoxin B_1_ and HD on hepatic redox status of broilers ^1^.

	Control	AFB_1_	AFB_1_ + 500HD	AFB_1_ + 1000HD
SOD, U/mg Protein	88.7 ± 12.1 ^ab^	83.7 ± 7.1 ^a^	94.5 ± 12.4 ^ab^	103 ±17.5 ^b^
GPX, U/mg Protein	26.2 ± 4.8 ^b^	21.0 ± 2.1 ^a^	24.0 ± 5.3 ^ab^	30.6 ± 7.8 ^b^
CAT, U/mg Protein	28.4 ± 5.1 ^ab^	25.9 ± 3.0 ^a^	27.5 ± 5.4 ^ab^	36.5 ± 9.9 ^b^
MDA, nmol/mg Protein	1.15 ± 0.26	1.01 ± 0.24	1.18 ± 0.22	1.25 ± 0.32
PC, nmol/mg Protein	6.19 ± 1.04 ^a^	8.26 ± 2.09 ^b^	6.08 ± 0.92 ^a^	5.58 ± 0.18 ^a^

^1^ Values are means ± SEs, *n* = 6. Labeled means in a row without a common letter differ, where *p* < 0.05. HD, *Hedyotis diffusa*; SOD, superoxide dismutase; GPX, glutathione peroxidase; CAT, catalase; MDA, malondialdehyde; PC, protein carbonyl; Control, base diet; AFB1, base diet supplement with 0.5 mg/kg AFB1; AFB1+500 HD, base diet supplement with 0.5 mg/kg AFB1 plus 500 mg/kg HD; AFB1+1000HD, base diet supplement with 0.5 mg/kg AFB1 plus 1000 mg/kg HD.

## Data Availability

Data are contained within the article.

## Supplementary Materials

The following are available online at https://www.mdpi.com/article/10.3390/antiox10060878/s1: Table S1: Basal diet formulation and nutritional values; Table S2: List of primers used for Q-PCR analysis; Table S3: Name, type, dilution, and source of primary antibodies.
