# Supplementary material for: Mitigation of Aflatoxin B1 Hepatoxicity by Dietary Hedyotis diffusa Is Associated with Activation of NRF2/ARE Signaling in Chicks
Source: Antioxidants (Basel). 2021 May 30;10(6):878. doi: 10.3390/antiox10060878 (PMC8229166; doi:10.3390/antiox10060878)
Supplement: Supplementary file 1 [file antioxidants-10-00878-s001.zip › antioxidants-1233467-supplementary.pdf]

Table S1. Basal diet formulation and nutritional values

Ingredients	Percentage (%)	Nutrition component	Content
Corn	58.2	Crude protein (%)	20.3
Wheat bran	2.0	Metabolizable energy (MJ/kg)	12.3
Soybean meal	30.0	Calcium (%)	1.0
Soybean oil	2.5	Available phosphorus (%)	0.43
Cottonseed meal	3.0	Methione (%)	0.60
$CaCO_3$	1.0	Methione+cystine (%)	0.90
$CaHPO_4$	2.0	Lysine (%)	1.10
Salt	0.3	Trytophan (%)	0.22
L-Lysine (%)	0.15	Threonine (%)	0.69
DL-Methione (%)	0.3		
Choline chloride	0.2		
Vitamin premix[1]	0.05		
Mineral premix[2]	0.3		

[1]Vitamin premix provided per kg of diet: retinyl acetate, 10280 IU; cholecalciferol 2280 IU; dl-α-tocopheryl acetate, 17.12 mg; menadione, 6.82 mg; thiamin, 2.28 mg; riboflavin, 5.68 mg; pantothenic acid, 12.25 mg; pyridoxine, 2.28 mg; niacin, 22.84 mg; biotin, 0.18 mg; folic acid, 1.12 mg.

[2]Mineral premix provided per kg of diet: iron, 100 mg; copper, 8 mg; manganese, 20 mg; zinc, 100 mg; iodine, 0.7 mg.

Table S2. List of primers used for Q-PCR analysis[1]

	Forward primer (5' to 3' direction)	Reverse primer (5' to 3' direction)
NRF2	ACGCTTTCTTCAGGGGTAGC	GTTCGGTGCAGAAGAGGTGA
NQO1	CGCACCCTGAGAAAACCTCT	AAGCACTCGGGGTTCTTGAG
HO1	GTCGTTGGCAAGAAGCATCC	GGGCCTTTTGGGCGATTTTC
GCLC	GGACGCTATGGGGTTTGGAA	AGGCCATCACAATGGGACAG
GSTA2	GATGGAGTCAATTCGGTGGC	GCACCATCTTCATCCCATCG
GSTA3	CACTATGCCAACACACGAGG	GGGCTCTCTCCTTCAGATCC
β-actin	ACCCCAAAGCCAACAGAG	CCAGAGTCCATCACAATACCAG

[1]*GCLC*, glutamate-cysteine ligase catalytic subunit; *GSTA2*, glutathione S-transferase alpha 2; *GSTA3*, glutathione S-transferase alpha 3; *HO1*, heme Oxygenase-1; *NQO1*, NAD(P)H: quinone oxidoreductase-1; *NRF2*, nuclear factor erythroid-2 related factor 2.

Table S3. Name, type, dilution, and source of primary antibodies[1]

Antibody	Isotype	Dilution	Source
Primary Antibody			
NRF2	Rabbit	1:1000	ABclonal (Wuhan, China)
NQO1	Rabbit	1:1000	ABclonal (Wuhan, China)
HO1	Rabbit	1:1000	ABclonal (Wuhan, China)
GCLC	Rabbit	1:1000	ABclonal (Wuhan, China)
GSTA2	Rabbit	1:1000	ABclonal (Wuhan, China)
GSTA3	Rabbit	1:1000	ABclonal (Wuhan, China)
β-actin	Rabbit	1:10000	ABclonal (Wuhan, China)
Secondary Antibody			
HRP labelled Antibody	Goat anti rabbit	1:10000	ABclonal (Wuhan, China)

[1]GCLC, glutamate-cysteine ligase catalytic subunit; GSTA2, glutathione S-transferase alpha 2; GSTA3, glutathione S-transferase alpha 3; HO1, heme Oxygenase-1; NQO1, NAD(P)H:quinone oxidoreductase-1; NRF2, nuclear factor erythroid-2 related fa
